# Appointing nurses trained in organ donation to improve family consent rates

**DOI:** 10.1111/nicc.12462

**Published:** 2019-07-11

**Authors:** Marloes Witjes, Nichon E. Jansen, Jacqueline van Dongen, Ingeborg H. F. Herold, Luuk Otterspoor, Bernadette J. J. M. Haase‐Kromwijk, Johannes G. van der Hoeven, Wilson F. Abdo

**Affiliations:** ^1^ Dutch Transplant Foundation Leiden The Netherlands; ^2^ Department of Intensive Care Medicine Radboud University Medical Center Nijmegen The Netherlands; ^3^ Department of Intensive Care Medicine Catharina Hospital Eindhoven The Netherlands

**Keywords:** consent rate, family guidance, nurses, organ donation

## Abstract

**Background:**

One of the most important bottlenecks in the organ donation process worldwide is the high family refusal rate.

**Aims and objectives:**

The main aim of this study was to examine whether family guidance by trained donation practitioners increased the family consent rate for organ donation.

**Design:**

This was a prospective intervention study.

**Methods:**

Intensive and coronary care unit nurses were trained in communication about donation (ie, trained donation practitioners) in two hospitals. The trained donation practitioners were appointed to guide the families of patients with a poor medical prognosis. When the patient became a potential donor, the trained donation practitioner was there to guide the family in making a well‐considered decision about donation. We compared the family consent rate for donation with and without the guidance of a trained donation practitioner.

**Results:**

The consent rate for donation with guidance by a trained donation practitioner was 58.8% (20/34), while the consent rate without guidance by a trained donation practitioner was 41.4% (41/99, *P* = 0.110) in those patients where the family had to decide on organ donation.

**Conclusions:**

Our data suggest that family guidance by a trained donation practitioner could benefit consent rates for organ donation.

**Relevance to clinical practice:**

Trained nurses play an important role in supporting the families of patients who became potential donors to guide them through the decision‐making process after organ donation request.

**WHAT IS KNOWN ABOUT THIS TOPIC**

- One of the most important bottlenecks in the organ donation process worldwide is the high family refusal rate.
- Long‐term contact between health care providers and families, in combination with training in donation practices, is associated with higher family consent rates.

**WHAT THIS PAPER ADDS**

- Guidance by a trained donation practitioner might lead to a higher consent rate.
- The implementation of trained donation practitioners is feasible, although 24/7 coverage is difficult to obtain without sufficient funding and larger‐scale training of nurses.
- It should be studied whether the late strategy is as effective as the early strategy when it comes to consent rates as the late strategy is easier and more cost‐effective to implement.

## 1 INTRODUCTION

In the Netherlands, with its opt‐in donor registration system, approximately 60% of the population is not registered in the national donor registry (DR). In these cases, donation is only allowed with the explicit consent of the next of kin (opt‐in consent system). The next‐of‐kin needs to make this important and difficult decision at a very emotional moment, which is one of the reasons why the national refusal rate is as high as 68% for potential donors who are not registered in the DR.^1^

## 2 BACKGROUND

With regard to requester characteristics and the communication processes in organ donation, the literature shows several ways to increase the consent rates: adequate information on brain death and the donation process,^2^, ^3^, ^4^ timing of the request,^3^ making the request in a private setting,^3^, ^4^ using trained and experienced individuals to make the request, and guiding the family through the decision‐making process.^3^, ^5^ In the United Kingdom, for example, a Specialist Nurse‐Organ Donation (SN‐OD) is involved from the moment it is apparent that life‐sustaining treatment will be withdrawn.^6^, ^7^ SN‐ODs are trained in communication and family support. Their role is to support potential donor families and the operational processes of organ donation. The advantage of the SN‐OD is that he or she had special training and has time to bond with the family and develop a relationship.

In the Netherlands, donation after brain death and (controlled) donation after circulatory death are being performed. The donation request is the responsibility of the treating physician, mostly an intensive care unit (ICU) physician. Since 2012, Dutch intensivists are obliged to complete the “communication about donation” (CaD) training. When the potential donor and/or family consents to organ donation, a transplant co‐ordinator becomes involved to co‐ordinate and supervise the organ donation procedure and to inform the family about the procedure.

The Dutch Transplant Foundation conducted a study in 2007 to 2009 with the aim of examining whether long‐term contact between health care providers and families, in combination with training in donation practices, was associated with higher consent rates. In this intervention study, three hospitals were compared, each using different approaches on this matter.^8^ The hospital that had ICU nurses who were trained in CaD to provide guidance to the relatives of potential donors had a higher consent rate for tissue and organ donation.

## 3 AIM OF THIS STUDY

Based on these previous results, the Dutch Ministry of Health allocated limited funding for two hospitals to train ICU nurses if these hospitals wanted to implement an approach where these trained nurses would provide guidance to relatives of potential donors. Our first aim was to study whether guidance by a trained donation practitioner (TDP) led to a higher family consent rate in hospitals that implemented such an approach. Because both hospitals used a different approach, our second aim was to compare the consent rates after implementing two different strategies.

## 4 METHODS

### 4.1 Study set‐up

The CaD training was developed by the Dutch Transplant Foundation in 2007 for physicians and nurses who are involved in family guidance of potential organ donors. The aim of the training was to improve communication skills and techniques, provide tools for discussing donation with relatives, give information about organ and tissue donation, and deal with different family reactions to the loss of a loved one. The training consisted of an e‐learning module that prepares the participant for a half‐day practical training in communication skills and techniques, including role play with actors.

ICU and coronary care unit (CCU) nurses were trained in CaD in two hospitals in the Netherlands (one university hospital and one general hospital). These nurses were designated as TDPs. In one hospital, patients with cardiac emergencies necessitating invasive mechanical ventilation could also be treated at the CCU, while in the other hospital, invasive mechanical ventilation was only possible in the ICU.

The guidance by a TDP was implemented in different ways in the two hospitals. In one hospital, an “early strategy” was used, which resembles the strategy used in the intervention hospital in the earlier study.^8^ The TDPs guided the families of patients admitted to the ICU with an acute intracerebral problem, Glasgow Coma Scale <8, and no contraindications for organ donation. These patients were selected because they had a higher risk of dying and becoming an organ donor because of their extensive brain injury. The rationale behind guidance of these families was that long‐term contact between a dedicated health care professional and the family would create more trust, thus benefitting organ donation consent rates. In the other hospital, a “late strategy” was used. TDPs guided families of patients in whom end‐of‐life care had started. Logistically, this approach was easier to implement as TDPs did not have to guide the family during the entire ICU admission but only from the moment the patient became a potential organ donor and organ donation was requested.

Potential patients were screened by the senior nurse for family guidance by a TDP. In both hospitals, the TDP participated in the family conversation about organ donation. After the conversation, the TDP had time to stay with the family, while the physicians and nurse returned to the department. This allowed the TDP to support the family, answer questions, and guide them through the decision‐making process after the organ donation request. In both hospitals, 25 nurses were trained. The hospitals were followed for 3 years from 2013 to 2016.

In the pilot study of Jansen et al,^8^ a TDP was available 24/7. Because of a lack of sufficient funding, we were unable to have a TDP standby 24/7, which resulted in the unavailability of a TDP in many donation requests. In both hospitals, we therefore chose the donation requests “without TDP” as the control group as they occurred in the same study period and hospitals.

### 4.2 Data analysis

First, we compared the family consent rate for donation “with guidance by a TDP” with the consent rate “without guidance by a TDP.” Second, we compared the consent rates of the two different strategies. This was performed by comparing the family consent rate with guidance by a TDP in the hospital with the early strategy with the hospital with the late strategy. The consent rates were reported with two‐sided *P*‐values. The significance level was set at *P* < 0.05.

We performed a Pearson Chi‐square test to test the differences in consent rates between guidance and no guidance by a TDP and the consent rate between the two strategies. Fisher's Exact test was used when one of the groups included fewer than 50 participants. The consent rates are shown, including potential donors who were registered with consent in the DR, as well as excluding consent in the DR. This was carried out to prevent an overestimation of the consent rate. The analyses were performed using SPSS (IBM), version 22.

## 5 ETHICAL AND RESEARCH APPROVALS

According to Dutch law, data generated by this study met the standard of exemption of the ethics board. In light of previous data, we aimed to set up a clinical improvement process where we used trained nurses. Ethically, our rationale was that additional family guidance could benefit families as they would not receive less but more guidance than normal practice. The most important aspect we considered was that the guidance would be given by trained nurses who already worked in the ICU or CCU.

## 6 RESULTS

**Figure 1:**
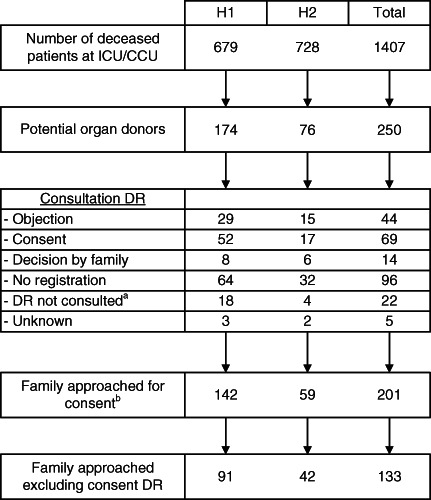
Flow diagram showing the inclusion of potential organ donors for each hospital and both hospitals together. ^a^Reasons for not consulting DR (n = 22): no Dutch nationality (n = 10), potential donor <12 years (n = 9), donor legally incapable (n = 1), objection according to relatives (n = 1), patient objected (n = 1). ^b^ Reasons for not approaching family for consent (n = 49): potential donor was registered with objection to donation (n = 44), family could not be reached (n = 3: DR not consulted (n = 2) and no registration (n = 1)), potential donor was not recognized as potential donor (n = 1: consent registration), unknown (n = 1: DR not consulted). CCU, coronary care unit; DR, donor registry; H1, hospital with early strategy; H2, hospital with late strategy; ICU, intensive care unit

Figure 1 shows the inclusion of potential organ donors for the hospitals separately and both hospitals together. A total of 1407 patients died in the ICU/CCU in both hospitals; 250 were potential organ donors (18%), and 201 families were approached to discuss donation.

### 6.1 Appointing TDPs

In the hospital with the early strategy, 142 family approaches for donation were made (Figure 1). In 25 cases, the family was guided by a TDP. In the other 117 cases, unavailability of a TDP prevented guidance. Because in this hospital TDPs were appointed to guide families at an early stage, they also guided families of patients who eventually never became a potential donor, that is did not die (n = 41, data not shown in Figure 1).

In the hospital with the late strategy, TDPs were appointed when end‐of‐life care started, and the organ donation request was made. In this hospital, 33 of 59 donation requests were guided by a TDP.

### 6.2 TDP vs no TDP

Table 1 shows the consent rates for donation requests with guidance by a TDP compared with donation requests without guidance by a TDP. We found higher consent rates when TDP guidance was applied. However, because of a lack of power, this did not result in statistical significant differences.

**Table 1:** Consent rates for donation requests; families guided by a trained donation practitioner (TDP) vs families without TDP guidance

	TDP n/n_total_ (%)	Without TDP n/n_total_ (%)	*P*‐value
All potential organ donors, excluding those with registered objection in the donor registry			
H1	17/25 (68.0)	72/117 (61.5)	0.651^a^
H2	24/33 (72.7)	11/26 (42.3)	0.032^a^
Total (H1 + H2)	41/58 (70.7)	83/143 (58.0)	0.095
All potential organ donors, excluding those registered with explicit consent or objection in the donor registry			
H1	7/13 (53.8)	35/78 (45.0)	0.565^a^
H2	13/21 (62.0)	6/21 (29.0)	0.062^a^
Total (H1 + H2)	20/34 (58.8)	41/99 (41.4)	0.110^a^

*Note*: H1, hospital with early strategy; H2, hospital with late strategy.

a Fisher's exact test.

Analysis of the donation requests with TDP did not show a statistically significant difference in consent rate between the hospital with the early strategy and the late strategy (68.0% vs 72.7%, *P* = 0.78). In addition, when we excluded potential donors registered with consent, there was no significant difference in consent rate between the early and late strategy (53.8% vs 62.0%, *P* = 0.73).

## 7 DISCUSSION

We found that guidance by a nurse as a TDP led to a higher consent rate, although this was not statistically significant as a result of a lack of power because of small sample size. Between the two different strategies, we also did not find a statistically significant differences in consent rate.

According to Jansen et al,^8^ the combination of training and long‐term contact increased consent rates. They, however, compared the hospital that used this “early strategy” with two control hospitals: one that employed hostesses who were not trained and another hospital without any type of guidance.^8^ It might be that guidance by trained TDPs without long‐term contact also increases consent rates as, in our results, we did not find a difference in consent rate between the hospitals using the early and late strategy. This would suggest that guidance by trained personnel could have a larger effect on consent rates than the duration of the guidance. Logistically, the late strategy is easier to implement as TDPs do not have to be present from the moment the patient is admitted to the ICU. In addition, with the early strategy, TDP guidance will often occur in patients who will survive and will not become a potential donor. Another difference from the study of Jansen et al is that, in the Jansen et al study, a TDP was available 24 hours a day for family guidance.

In the United Kingdom and United States, the organ procurement staff is involved in the organ donation requests.^9^, ^10^, ^11^ In the United States, well‐trained specialized organ procurement organization (OPO) co‐ordinators screen for medical suitability, perform the donation request, and co‐ordinate allocation and recovery of organs while providing emotional support to families. The OPO works closely together with the treating team, but it is the OPO co‐ordinator who takes the lead in the conversations with the family regarding organ donation and transplantation.^12^ In the United Kingdom, the standard of best practice is a collaborative family approach between the senior medical staff and the SN‐OD.^7^ This is not the practice in the Netherlands, where the ICU physician usually performs the donation request. In the Netherlands, the transplant co‐ordinator becomes involved in the family conversations after family consent to organ donation has been obtained. A study by Hulme et al. showed that involvement of a specialist nurse is associated with a higher consent rate, with an even stronger association when the specialist nurse led the conversation about donation.^11^ A study in Sweden also showed that working with trained nurses called “Donation Specialist Nurses” increased the number of eligible donors who became actual donors from 37% to 74%, mostly because of an increased family consent rate.^13^ An earlier study, the ACRE trial in the United Kingdom,^14^ which was performed after the implementation of SN‐ODs, showed no effect of collaborative requesting on the consent rate. Collaborative requesting means that the relatives are approached by the clinical team and a donor transplant co‐ordinator together. However they did not define the roles of the physician and donor transplant co‐ordinator during the family approach. In addition, the transplant co‐ordinators were not trained in making the actual donation request, while the specialist nurses in the study of Hulme et al. received training in communication and family support.

In the Spanish model, the transplant co‐ordinators are in‐house professionals who are staff members of the procurement hospital. The majority of the transplant co‐ordinators are critical care physicians.^15^ This creates a situation that guarantees proper donor identification. Most studies have shown that the involvement of organ procurement staff, or nurses, in the family approach increases the consent rate.^10^, ^11^, ^16^, ^17^ Whether the request is performed by a clinician, organ procurement representative, or nurse, the literature is consistent that the requester should be trained.^4^, ^5^, ^18^, ^19^, ^20^

## 8 LIMITATIONS

We expected to find higher consent rates with guidance of a TDP. In addition, we expected a larger effect in the early strategy compared with the late strategy. We were unable to show these effects possibly because of two reasons. First, the sample size of our study was relatively small. Because of lack of funding, there were not enough trained TDPs to cover donation requests on a 24/7 basis. This was especially difficult in acute situations, weekends, or in the nights. Second, selection bias may have occurred. In this study, we focused on the consent rate as an outcome measure, but confounding variables that could have influenced consent rate were not measured or controlled for: for example, age and sex of potential organ donor, hospital length of stay, known donation wishes of potential donor, family knowledge and attitudes about donation, circumstances of death, and time of the day request was made.^9^, ^11^, ^16^, ^21^

## 9 IMPLICATIONS AND RECOMMENDATIONS FOR PRACTICE

Considering our results and the results from previous studies, we recommend implementing guidance by TDPs in more hospitals. In addition, it should be studied whether the late strategy is as effective as the early strategy as the late strategy is easier and more cost‐effective to implement. Based on our results, 167 patients per group would be needed to test whether the late strategy would be inferior to an early guidance strategy (power 0.80, alpha 0.05, difference in consent rate of 15%).

In the hospital with the early strategy, families of potential organ donors are still being guided by a TDP if a TDP is available. In the hospital with the late strategy, another initiative has been developed. In this hospital, all patients admitted to the ICU with a poor medical prognosis and an expected hospital stay of longer than 72 hours receive additional guidance from a nurse. The rationale behind this is that additional guidance in the ICU is important and beneficial to all families irrespective of organ donation. However, these nurses guiding the families are not all trained in CaD. What we have noticed in the two hospitals we studied was that implementing TDPs was more difficult to realize when the total pool of ICU nurses was large (ie, University hospital) as training of a large pool of nurses would be needed to cover a larger amount of donation requests. This problem can partially be addressed if such training is made part of the regular education and training of ICU nurses. Another solution could also be to involve the already existing transplant co‐ordinator earlier in the donation process. Instead of involving the transplant co‐ordinator after consent had been given, the transplant co‐ordinator could also be involved before or during the donation request.

## 10 CONCLUSION

Guidance by a nurse as a TDP could lead to a higher family consent rate, although we did not find a statistically significant effect because of small sample size. Future research could shed more light on which strategy to guide families would be most feasible to implement nationally.
